# Supplementary material for: A Proteomics-Based Approach Identifies the NEDD4 Adaptor NDFIP2 as an Important Regulator of Ifitm3 Levels
Source: Viruses. 2023 Sep 26;15(10):1993. doi: 10.3390/v15101993 (PMC10611234; doi:10.3390/v15101993)
Supplement: Supplementary file 1 [file viruses-15-01993-s001.zip › Supplementary Figures revised.pdf]

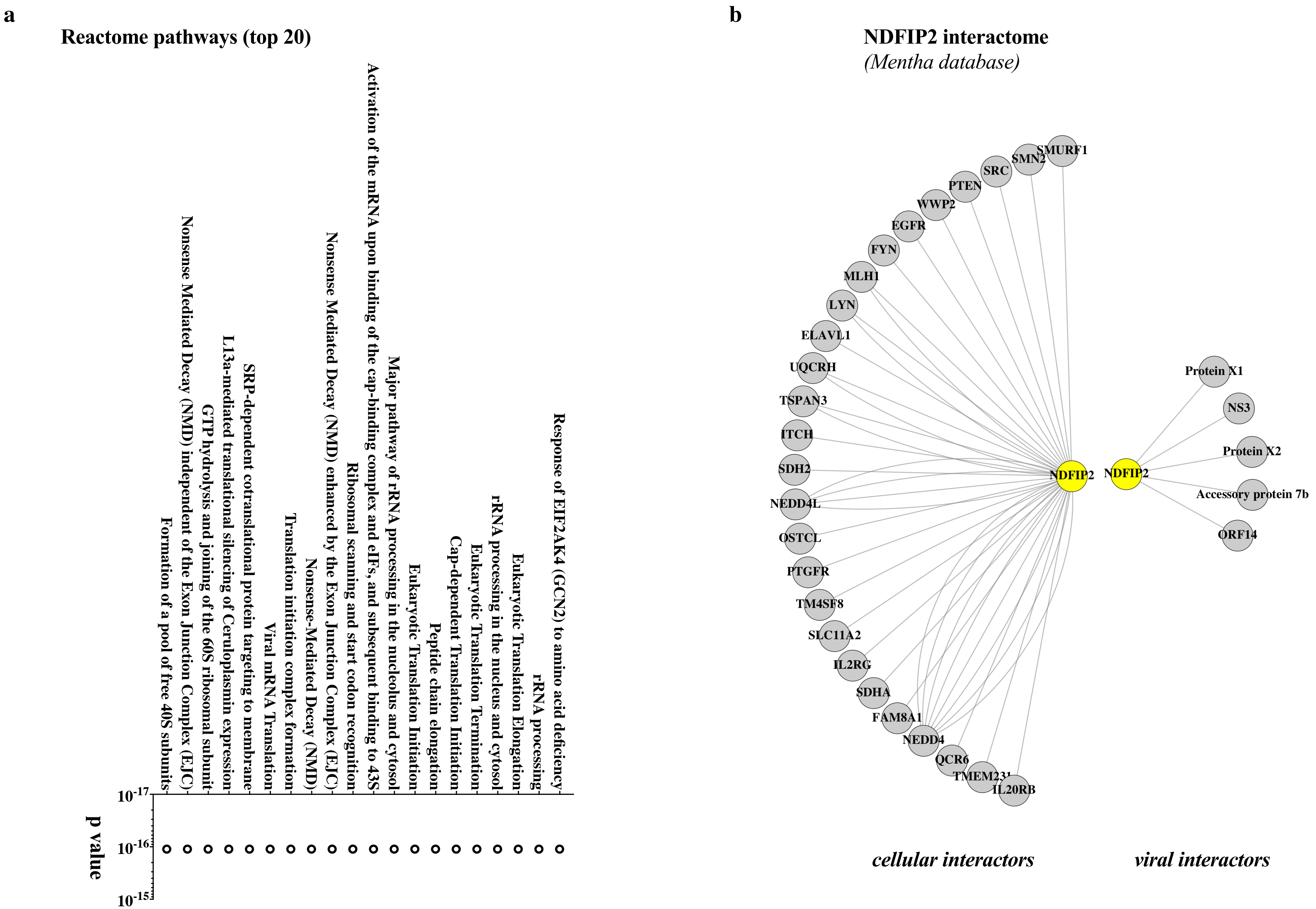

Figure S1. Reactome pathways analysis of proteins enriched in IFITM3-exosomal fractions. a) The graph represents the top 20 Ingenuity pathways identified among proteins differentially incorporated in IFITM3- over control-exosomal fractions. **b**) Analysis of the interactome of NDFIP2 (Mentha database).

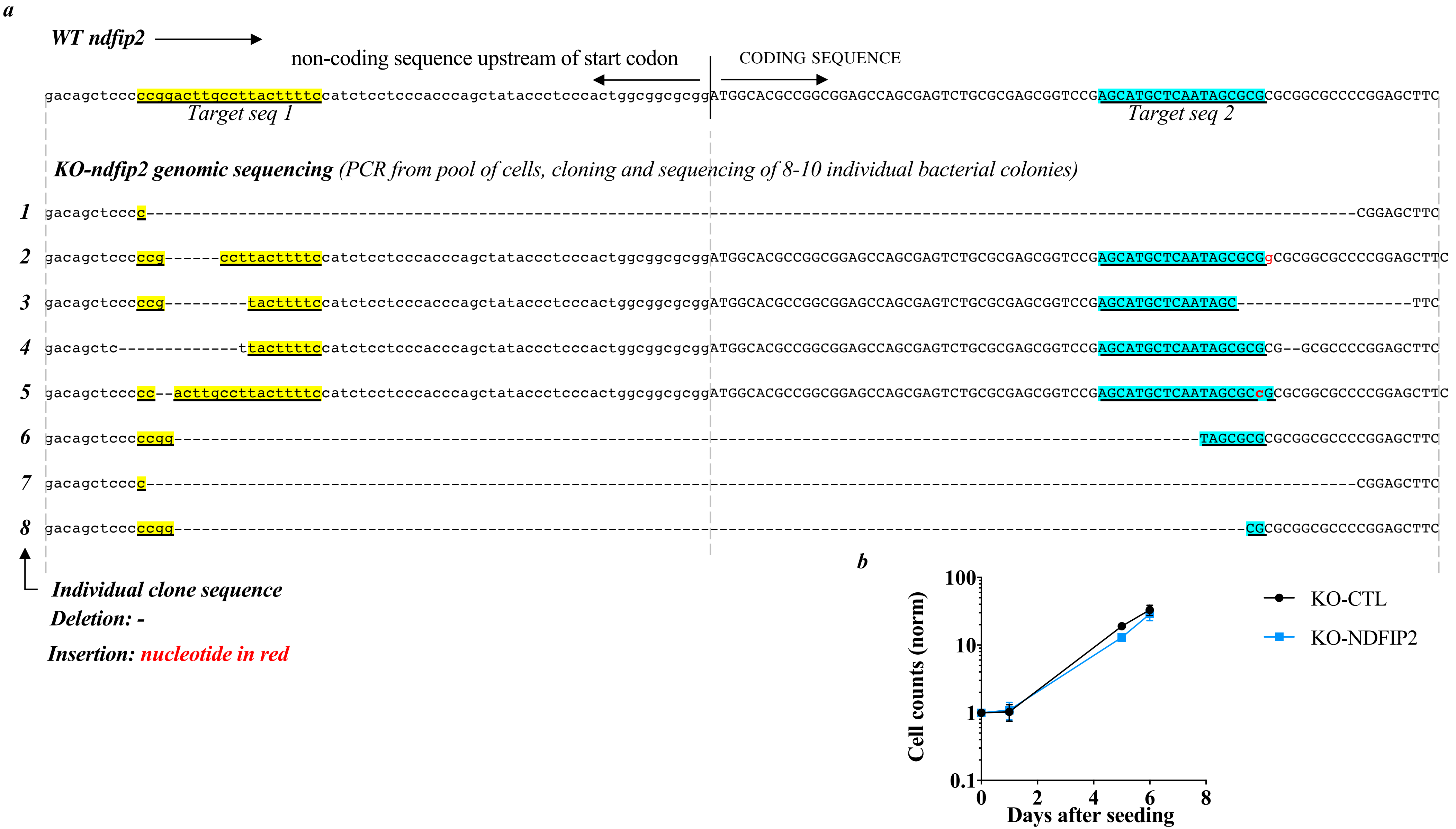

Figure S2. Characterization of A549 epithelial lung cells knocked out for NDFIP2. Pools of control or *ndfip2* A549 genetic KO cells were generated upon lentiviral-mediated gene transduction of CRISPR/Cas9 and selection. a) Genomic DNA was extracted and the region spanning the CRISPR target sequences was PCR-amplified, cloned and individual colonies sequenced. The sequences obtained after amplification from CRISPR control cells were *wild-type* and are thus not shown here for simplicity. b) Cells were seeded and counted at the indicated time points to estimate the cell doubling time. No effects of ndfip2 deletion were observed under these experimental conditions (n=3).

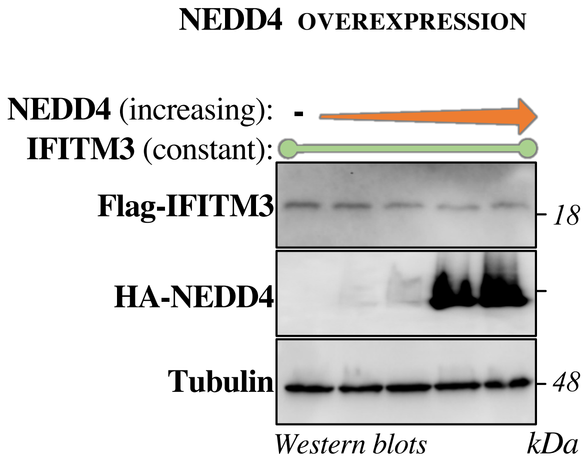

Figure S3. NEDD4 overexpression does not lead to modifications of the steady-state levels of IFITM3 in HEK293T cells. HEK293T cells were transfected with a constant amount of DNA coding Flag-IFITM3 together with increasing doses of DNA coding HA-NEDD4 (0.1 µG of IFITM3 and from 0.05 to 0.4 µG of HA-NEDD4 coding plasmid, in 24-well plates), prior to WB analysis twenty-four hours post transfection, in the same experimental conditions of the experiment presented in Fig. 2b. The WB panels present typical results obtained (n>3).

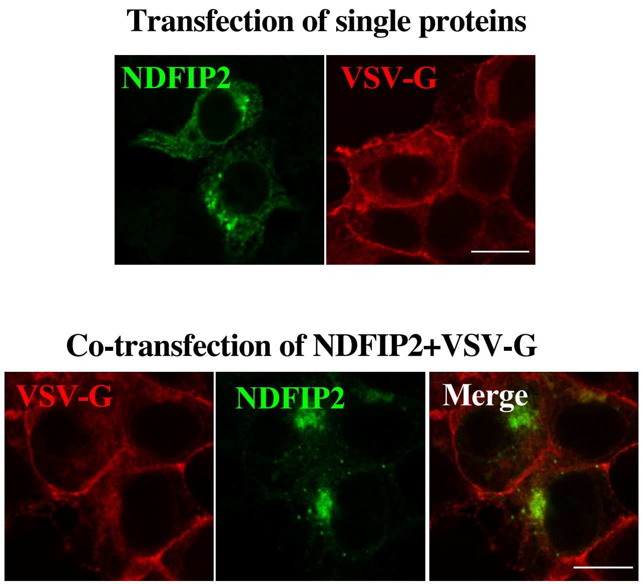

Figure S4. Lack of specific relocalization of VSV-G upon co-expression of NDFIP2. HEK293T cells transfected with DNAs coding NDFIP2 alone or together with the protein G of the Vesicular Stomatitis Virus (VSV-G) used here as control protein unrelated to IFITM3. The pictures present typical results obtained.
